# Draft Genome Sequence of Escherichia coli Phage CMSTMSU, Isolated from Shrimp Farm Effluent Water

**DOI:** 10.1128/MRA.01034-18

**Published:** 2018-10-11

**Authors:** Lelin Chinnadurai, Thirumalaikumar Eswaramoorthy, Abinaya Paramachandran, Sayan Paul, Rashmi Rathy, Arun Arumugaperumal, Sudhakar Sivasubramaniam, Citarasu Thavasimuthu

**Affiliations:** a Centre for Marine Science and Technology, Manonmaniam Sundaranar University at Rajakkamangalam, Kanyakumari, Tamilnadu, India; b Department of Biotechnology, Manonmaniam Sundaranar University, Tirunelveli, Tamilnadu, India; Indiana University Bloomington

## Abstract

The Escherichia coli phage CMSTMSU was isolated from shrimp farm effluent water in Ramanathapuram, India. The phage exhibited lytic activity against both E. coli and the fish pathogen Pseudomonas aeruginosa. Here we report the draft genome sequence, assembly, and annotation of the isolated CMSTMSU phage. This genome resource can be used to utilize the phage as a crucial biocontrol agent in the fish aquaculture sector.

## Abstract (precis)

The Escherichia coli phage CMSTMSU was isolated from shrimp farm effluent water in Ramanathapuram, India. The phage exhibited lytic activity against both E. coli and the fish pathogen Pseudomonas aeruginosa.

## ANNOUNCEMENT

Bacteriophages are viruses that infect bacteria. They are abundant in natural systems and are considered crucial factors in controlling bacterial populations (1). Phages also have the potential to regulate bacterial diseases of fish in aquatic environments by removing the fish pathogens (2). This study reports the genome sequence, assembly, and annotation of the Escherichia coli phage CMSTMSU. The phage was isolated from a wastewater sample obtained from a shrimp farm located in Ramanathapuram, India. It was detected with the soft agar overlay method using log-phase E. coli cells as the host. The isolated CMSTMSU phage also exhibited lytic activity against the fish pathogen Pseudomonas aeruginosa.

The E. coli phage CMSTMSU was purified following the protocol reported by Mullan (see https://www.dairyscience.info/index.php/isolation-and-purification-of-bacteriophages.html). Then, the genomic DNA was extracted with the phenol-chloroform extraction method (3). The DNA library was prepared with the NEBNext Ultra II DNA library prep kit (New England Biolabs, USA). The whole-genome sequencing was performed with MinION Mk1b (Oxford Nanopore Technologies, UK) using the SpotON flow cell (FLO-MIN106) (4), and base calling was performed with Albacore version 2.1.3 at Genotypic Technology Pvt Ltd (Bangalore, India). We obtained 88,676 reads from the bar-coded library with the Nanopore sequencer with an average read length of 3.4 kb and an *N*_50_ length of 6,531 bp. The quality of the reads was analyzed with FastQC software version 0.11.5 (5). The base-called raw reads were used for *de novo* assembly with the Canu algorithm (6). The Canu assembly generated a single contig of 386.4 kb, which has a GC content of 35.6%. The contig underwent a BLAST search against the NCBI virus nonredundant (nr) database with the BLASTN algorithm with an E value threshold of 1E-5, and we found that it has an 83% sequence similarity with Escherichia phages PBECO 4, vB_Eco_slurp01, and 121Q.

The draft genome of E. coli phage CMSTMSU was annotated with the RAST annotation server version 2.0 (http://rast.nmpdr.org) (7), GeneMarkS version 4.28 (http://exon.gatech.edu/GeneMark/genemarks.cgi) (8), and GLIMMER version 3.02 (https://ccb.jhu.edu/software/glimmer/) (9) gene prediction tools. The data obtained from the RAST annotation identified 767 protein-coding genes, and among them, 715 (91%) genes were identified from a BLAST search against the NCBI virus database with the BLASTP algorithm. The gene ontology (GO) and KEGG pathway annotations of the protein-coding genes were performed with the Blast2GO (https://www.blast2go.com/) functional annotation software (10). Of the 715 BLAST-annotated genes, 190 genes were assigned to 423 GO terms with ATP binding (45 genes) and nucleic acid phosphodiester bond hydrolysis (32 genes), and these were the most highly represented GO terms in the data set. We mapped 117 genes with 12 KEGG metabolic pathways, among which the pathways associated with purine metabolism (37 genes) and pyrimidine metabolism (26 genes) were the most dominant in the genome data set. Simultaneously, the annotations with the GeneMarkS and GLIMMER gene prediction tools predicted 891 and 938 protein-coding genes, respectively. Among these predicted genes, 599 genes were common to all three databases, whereas 115, 12, and 115 genes showed an overlap between RAST and GLIMMER, RAST and GeneMarkS, and GeneMarkS and GLIMMER, respectively. In addition, we identified 6 tRNA genes with a GC content range from 48.6% to 58.4% with the ARAGORN version 1.2.38 program (11). This genome draft sequence can be used as a potential resource to utilize the phage species as a biocontrol agent of antibiotics against fish pathogens.

### Data availability.

The raw sequence reads have been submitted to the NCBI SRA under the accession number SRP158495, and the draft genome sequence of Escherichia coli phage CMSTMSU has been deposited in NCBI GenBank under the accession number MH494197.
